# Characteristics of Women, Intrapartum Interventions, and Maternal and Neonatal Outcomes Among Users of Intrapartum Water Immersion: The UK POOL Cohort Study

**DOI:** 10.1111/birt.12921

**Published:** 2025-05-12

**Authors:** Julia Sanders, Christy Barlow, Peter Brocklehurst, Rebecca Cannings‐John, Susan Channon, Judith Cutter, Billie Hunter, Mervi Jokinen, Fiona Lugg‐Widger, Sarah Milosevic, Chris Gale, Rebecca Milton, Leah Morantz, Shantini Paranjothy, Rachel Plachcinski, Michael Robling

**Affiliations:** ^1^ School of Healthcare Sciences Cardiff University Cardiff UK; ^2^ Centre for Trials Research Cardiff University Cardiff UK; ^3^ Birmingham Clinical Trials Unit University of Birmingham Birmingham UK; ^4^ Cardiff and Vale University Health Board Cardiff UK; ^5^ Royal College of Midwives London UK; ^6^ Neonatal Unit Chelsea and Westminster NHS Foundation Trust London UK; ^7^ Neonatal Medicine, School of Public Health, Faculty of Medicine Imperial College London London UK; ^8^ Centre for Paediatrics and Child Health Imperial College London UK; ^9^ Public and Patient Representative; ^10^ School of Medicine Institute of Applied Health Sciences Aberdeen UK; ^11^ DECIPHer Cardiff University Cardiff UK

**Keywords:** intrapartum, intrapartum care, midwifery, water immersion, waterbirth

## Abstract

**Background:**

The POOL study explored intrapartum water immersion and associated maternal and neonatal outcomes at 26 UK sites 2015–2022.

**Methods:**

Retrospective and prospective data captured in electronic maternity and neonatal UK National Health Service (NHS) information systems. Analysis—(a) proportions of women using and factors associated with water immersion during labour or birth; (b) outcomes among “low‐risk” women who used water immersion during labour or birth; (c) management and outcomes of the third stage of labour following waterbirth.

**Results:**

Among 869,744 included births, 10% (*n* = 87,040) used water immersion during labour or birth and 4.6% (*n* = 39,627) gave birth in water, with rates falling over time. Being of white or multi‐ethnicity, fluent in English, non‐smokers or ex‐smokers, from more affluent areas, and nulliparous were associated with higher rates of water use. Overall, 39.6% of nulliparous and 9.9% of parous women at low risk at labour onset, and who used water immersion during labour, received obstetric or anesthetic care during the intrapartum period. Physiological third stage management was used following 27.1% (*n* = 10,737) of waterbirths and following 8.6% (*n* = 2260) of waterbirths the placenta was delivered into water. The rate of recorded blood loss ≥ 1000 mL was not significantly different when the placenta was delivered in water compared to placental delivery out of water.

**Conclusion:**

This large UK study of water immersion during labour and birth provides important information for policymakers, maternity health professionals, and for women and families considering the option of intrapartum water immersion. Care providers need to ensure equal access to intrapartum water immersion across demographic groups and provide women with evidence‐based rates of obstetric interventions that take into account their risk status and birth choices.

**Trial Registration:** ISRCTN13315580

## Graphical abstract

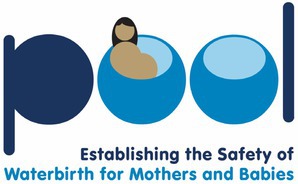

## 1 Introduction

The UK National Institute for Health and Care Excellence (NICE) recommended that women without pregnancy risk factors should be offered intrapartum water immersion analgesia but consider there is insufficient evidence to support birth in water [1, 2, 3]. Reports of adverse neonatal outcomes following waterbirth including water inhalation, sepsis, and cord avulion [4], increased rate of maternal Obstetric Anal Sphincter Injury (OASI) [5] and the inability to conduct randomized trials of waterbirth [6] contributed to continuing clinical concerns in the UK.

The primary objective of the POOL study was to determine for “low‐risk” women who used intrapartum water immersion and had an uncomplicated labour, whether waterbirth was as safe women and their babies as leaving the water before birth. The primary analysis included 73,229 births. Rates of OASI were no higher among nulliparous or parous women who had waterbirths compared to those who gave birth out of water. The rates of a composite adverse neonatal outcome (including death, the need for respiratory support in a neonatal unit, or the administration of antibiotics within 48 h of birth) were also no higher among waterbirths. The protocol [4] and primary study results [5] have been previously reported.

To enable women to make informed choices around intrapartum water immersion, they need availability to current, geographically relevant rates of transfer to obstetric care, the rates of obstetric interventions during labour, and birth outcomes. Additionally, to raise awareness of potential biases in the offering or uptake of water immersion during labour, it is essential to consider the characteristics of the women accessing water immersion. These data have previously been reported in the UK [6, 7] but more up‐toto‐date information was required.

In the UK, third stage management of midwives following waterbirth was poorly described, as were outcomes associated with placental delivery into water. A systematic review of 36 studies into waterbirth published before June 2021 [8] identified just one study including 51 cases with placental delivery in water, but without reporting outcomes [9]. More recent large studies of waterbirth [10, 11] have not reported outcomes associated with placenta delivery into water, and the funder requested that outcomes following placental delivery in water be described.

## 2 Objectives

This paper reports analysis of a priori objectives of the POOL study cohort: (a) the proportion of women giving birth using, and factors associated with, water immersion during labour or birth; (b) maternal and neonatal outcomes among all “low risk” women who used water immersion during labour; and (c) management and outcomes of the third stage of labour following waterbirth.

## 3 Methods

This cohort study used retrospective and prospective data captured in electronic maternity and neonatal UK National Health Service (NHS) information systems.

### 3.1 Setting and Participants

All 26 NHS sites that used the Euroking Maternity Information System (MIS) in England and Wales participated (Figure S1).

All sites provided obstetric, neonatal, and home birth services and had a main delivery suite providing intrapartum care. In addition, 23 had a Midwifery Led Unit (MLU) within the same hospital as the obstetric unit, of which five also had an MLU on a separate site from the obstetric unit. One had an obstetric unit and an MLU on separate sites. In keeping with many in the UK, some services temporarily reconfigured services during the Covid‐19 pandemic, reducing access to MLUs or home birth services [12]. Water immersion during labour or birth was recorded in electronic records by midwives and included immersion in a domestic bath or any birthing pool. Births in obstetric units, at home, and in MLUs were included. Births in which the fetus was partially born into water, including in the event of shoulder dystocia, a previously unrecognized breech presentation, or a woman standing between the birth of the fetal head and body, remained in the waterbirth group for the purposes of analysis. Births at which a midwife was not in attendance, either because the woman chose to give birth without professional assistance, or because birth occurred at home or elsewhere before professional assistance arrived or could be reached, were excluded.

In the UK it is usual that women without pregnancy complications in spontaneous labour receive intrapartum care from midwives, being referred to an obstetrician if complications develop or epidural analgesia is requested. Women were categorized as “low‐risk” and under midwifery care, if there was no record of complicating factors in their notes to suggest that birth should have been recommended to be planned in an obstetric unit [2] (Tables S1.1–S1.4, and S2). Among women commencing labour under midwifery care and using water immersion, referral to obstetric care was assumed to have occurred when interventions outside of midwifery practice in the UK, were recorded as having been provided. These included administration of intravenous Oxytocin for augmentation of labour, epidural analgesia, suspected sepsis, instrumental birth or caesarean section, manual removal of placenta or OASI repair. Management of the third stage of labour was categorized by the midwife providing care as “physiological” or “active.”

Births from January 1st 2015 to June 30th 2022 were included. Some data fields that were required to meet study objectives but were not collected within existing systems were added to the maternity information system at each study site, and these data were collected prospectively from the date individual sites opened. Where fields were collected prior to site opening, a combination of retrospective and prospective data were included in analyses. Duplicate records, blank records, and births before 24 weeks gestation were excluded.

### 3.2 Ethics Committee Approval

Research Ethics Committee (REC) approval was granted by the REC for Wales (18/WA/0291) and the transfer and use of identifiable data was approved by the Health Research Authority (HRA) Confidentiality Advisory Group (CAG) (18/CAG/0153). No consent was required from participants, but women in the prospective cohort could request not to be included in the study.

### 3.3 Data Collection

Data were collected in local electronic Maternity Information Systems at participating sites. Details of data collection procedures are fully described elsewhere [4, 5, 13]. Data relating to all babies born at study sites throughout this period who were admitted to a neonatal unit following maternal water immersion during labour were obtained from the National Neonatal Research Database [14].

### 3.4 Analysis/Outcomes

Analysis was performed to meet each objective (a): proportions of women using, and factors associated with, water immersion for labour or birth were described for the whole cohort, for 2016 when rates of water immersion were highest, and the last 12 months of data collection in 2021–2022. The number and rates of water immersion in labour and waterbirths per 100 births were described by calendar quarter, across the study period, and throughout the study period across study sites. We characterized women who used, or did not use, water immersion during labour and those that gave birth in water. Summary statistics were used depending on the data type, that is, numbers alongside percentages were used for categories and mean alongside standard deviation to describe continuous data such as gestational age (years).

(b): Maternal and neonatal outcomes among all “low‐risk” women, who used water immersion during labour or birth were explored by parity groups: nulliparous or parous (second, third or fourth birth). The rates of interventions (including augmentation with Oxytocin, regional analgesia or anesthesia, and modes of birth), and maternal outcomes (e.g., Obstetric Anal Sphincter Injury by mode of birth, postpartum hemorrhage) and adverse neonatal outcomes were described by parity group.

(c): The management and outcomes of the third stage of labour were examined among women who had a waterbirth, including management of the third stage of labour and postpartum obstetric involvement in care. Given the clinical uncertainty around appropriate management of the third stage of labour following waterbirth, the rate of recorded PPH of ≥ 1000 mL was tested between women who delivered their placenta in water and women who left the water during the third stage, with parameter estimate reported as an adjusted odds ratio alongside a two‐sided 95% confidence interval. Confounders adjusted for were (a) those associated with risk of postpartum hemorrhage: maternal age, parity, gestation, BMI, birthweight [1]; (b) those associated with anemia or circulating volume: ethnic group [15], deprivation quintile; (c) year and quarter of birth due to the potential for PPH management to have changed over time; and (d) recorded midwife concerns present prior to birth as this may result in a lower threshold for requesting a woman to leave the pool during the third stage. Additionally, the models allowed for clustering of outcomes within NHS site. Data analysis was conducted in IBM SPSS Statistics version 26 (IBM Corporation, Armonk, NY, USA) and Stata version 16 (StataCorp LP, College Station, TX, USA).

### 3.5 Patient and Public Involvement

Two members of the co‐investigators group were parent representatives, with lived experience of birth and waterbirth, and expertise in communicating with expectant parents. They were equal members of the study management group throughout the development and delivery of the study.

### 3.6 Role of Funding Source

The funder of the study had no role in the design, data collection, data analysis, data interpretation, or writing of the report.

## 4 Results

### 4.1 Water Immersion Use During Labour

**FIGURE 1:**
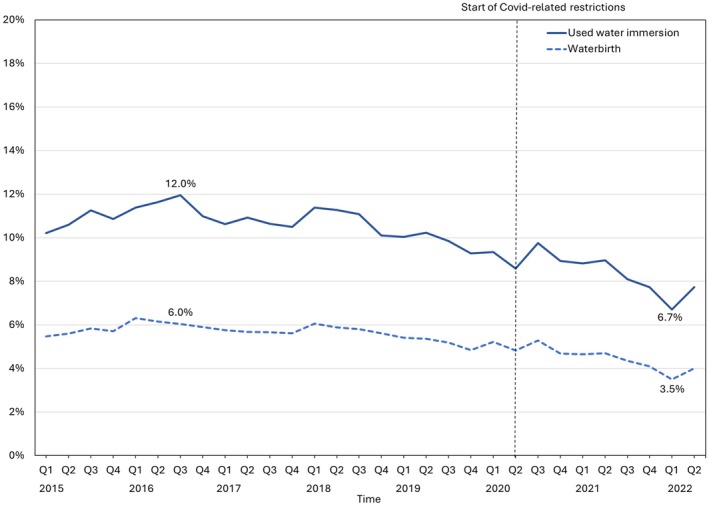
Rates of water immersion and waterbirth (based on all births at sites) across the study period. Vertical line indicates the start of the Covid‐19 pandemic; Q2 = April–June 2020. Based on *n* = 26 sites; *n* = 869,744 birth records; *n* = 87,040 using water immersion; 39,627 waterbirths. [Colour figure can be viewed at wileyonlinelibrary.com]

A total of 956,307 records were received. After exclusions, 10% of records (*N* = 87,040) indicated women had used water immersion during labour, with 39,627 (4.6%) women giving birth in water (Figure S2). The proportion of women using water during labour or birth was highest in quarter 3 (July–September), 2016 with rates of 12% and 6%, and lowest in quarter 1 (January–March) 2022 at 6.7% and 3.5% respectively (Figure 1). Variation was observed in the rate of women using water immersion during labour across the study sites (ranging from 23% to 2%) and in the rate of waterbirths (from 11% to 1%) (Figure S3).

The proportion of women in each category who used water during labour or birth and those that did not, are presented in Table 1. A lower proportion of women from an Asian or Asian British ethnic background; a Black, Black British, Caribbean, or African background; or from “other” ethnic groups; used water in labour than from a mixed or multiple ethnic groups; or white background (3.6%, 4.1%, 7.4%, 9.5%, 11.4%, respectively). A higher proportion of non‐smokers, or ex‐smokers used water than smokers (10.5%, 10.0% vs. 6.3% respectively) as did women with no recorded problems of issues with English language or literacy compared to women with no or limited ability to understand the English language (10.6% vs. 2.8% respectively). Women who used water immersion were more likely to reside in more affluent areas than women who did not use water immersion (least deprived quintile 13.1% vs. most deprived quintile 6.1% respectively) and be aged under 35 years. First time mothers were more likely to use water than multiparous women (11.8% vs. 8.7% respectively). Between 2016 and the last 12 months of data collection in 2021/2022 the proportions of women using water during labour reduced from 11.6% to 7.6% and waterbirths birth decreased from 8.2% to 3.2%. Reductions in water use and waterbirths reduced over this time period across all groups examined.

**TABLE 1:** Number and proportions of women in each category who used water during labor or birth 2016–2021/22.

Maternal characteristics	All women 2015–2022 *N* = 869,744		All women 2016^a^ *N* = 129,818		All women 2021/22 *N* = 114,588	
	Used water^b^	Waterbirth	Used water^b^	Waterbirth	Used water^b^	Waterbirth
Number of women	87,040 (10.0)	39,627 (4.6)	13,776 (11.5)	9799 (8.2)	8407 (7.6)	3572 (3.2)
Age category						
< 20 years	2395 (10.4)	863 (3.8)	436 (12.1)	303 (8.4)	137 (5.9)	44 (1.9)
20–24 years	12,072 (10.2)	5071 (4.3)	2172 (12.4)	1610 (9.2)	864 (6.6)	341 (2.6)
25–29 years	25,821 (10.8)	11,647 (4.9)	4221 (12.4)	3047 (9.0)	2262 (7.8)	913 (3.1)
30–34 years	31,331 (10.9)	14,632 (5.1)	4649 (12.2)	3285 (8.6)	3382 (8.7)	1454 (3.7)
35–39 years	14,003 (8.6)	6828 (4.2)	2097 (9.7)	1435 (6.6)	1600 (7.2)	758 (3.4)
40+ years	1418 (3.6)	586 (1.5)	201 (4.1)	119 (2.4)	162 (2.9)	62 (1.1)
Maternal ethnic group^c^						
White (inc. English, Welsh, Scottish, Northern Irish or British, Irish, Gypsy or Irish Traveler, Roma, any other)	70,484 (11.4)	32,420 (5.2)	11,211 (13.0)	7986 (9.2)	6899 (8.9)	2987 (3.8)
Asian or Asian British (inc. Indian, Pakistani, Bangladeshi, Chinese, any other)	3669 (3.6)	1643 (1.6)	593 (4.4)	418 (3.1)	338 (2.4)	119 (0.8)
Black, Black British, Caribbean or African (inc. any other)	1360 (4.1)	686 (2.1)	235 (5.1)	184 (4.0)	130 (2.9)	59 (1.3)
Mixed or multiple ethnic groups (White and Black Caribbean, African, White and Asian, inc. any other)	1242 (9.5)	595 (4.5)	159 (10.0)	121 (7.6)	153 (7.6)	66 (3.3)
Other ethnic group (inc. Arab and any other)	2544 (7.4)	1096 (3.2)	501 (9.9)	326 (6.5)	172 (4.2)	61 (1.5)
Not recorded	7741	3187	1077	764	715	280
Mother a smoker at booking						
Yes (including current use of e‐cigarettes)	6079 (6.3)	2529 (2.6)	1121 (8.4)	834 (6.2)	429 (3.8)	184 (1.6)
Ex‐smoker (stopped before/during pregnancy)	7532 (10.0)	3094 (4.1)	1069 (10.9)	774 (7.9)	589 (6.5)	220 (2.4)
Non‐smokers	73,429 (10.5)	34,004 (4.9)	11,586 (12.0)	8191 (8.5)	7389 (8.1)	3168 (3.5)
Understanding of English language						
No issues	73,962 (10.6)	32,636 (4.7)	10,399 (11.8)	7213 (8.2)	7896 (7.9)	3366 (3.4)
Limited/no ability to understand, speak or read English	1527 (2.8)	608 (1.1)	246 (4.6)	182 (3.4)	131 (1.9)	41 (0.6)
Not recorded	11,551	6383	3131	2404	380	165
Townsend deprivation quintile						
1—least deprived (most affluent)	18,011 (13.1)	8605 (6.3)	2797 (14.5)	1961 (10.1)	1834 (10.8)	837 (5.0)
2	20,185 (13.0)	9435 (6.1)	3105 (14.5)	2195 (10.2)	2042 (10.4)	893 (4.6)
3	18,913 (11.2)	8488 (5.0)	2810 (12.3)	2015 (8.9)	1900 (8.8)	782 (3.6)
4	15,727 (8.8)	6905 (3.8)	2708 (10.8)	1887 (7.5)	1403 (6.3)	597 (2.7)
5—most deprived (least affluent)	12,572 (6.1)	5475 (2.7)	2150 (7.5)	1580 (5.5)	974 (3.8)	362 (1.4)
Not recorded	1635	719	206	161	254	101
Parity at booking						
Nulliparous (parity 0)	44,045 (11.8)	15,176 (4.1)	7203 (14.1)	4552 (8.9)	4035 (8.4)	1308 (2.7)
Multiparous (parity 1+)	42,995 (8.7)	24,451 (4.9)	6573 (9.5)	5247 (7.6)	4372 (6.9)	2264 (3.6)

*Note:* All data are *n* (%) unless otherwise stated. To provide the proportion of women with each characteristic using water immersion or experiencing waterbirth percentages are based on the total number of women within the specific strata, for example, number of women age < 20 years using water divided by the number of women age < 20 years.

^a^ Period covered the most common use.

^b^ Excluding unattended births, duplicates, and records with no valid data.

^c^ Following Office National Statistics categories List of ethnic groups—GOV.UK (https://www.ethnicity‐facts‐figures.service.gov.uk/style‐guide/ethnic‐groups/).

Among the 87,040 women who used water immersion in labour, 13,811 (15.9%) were identified to have recorded medical or obstetric ‘risk‐factors’ at the time of commencing water immersion. Characteristics and outcomes among women with known ‘risk‐factors’ who used water immersion during labour will be reported separately.

### 4.2 Labour Interventions and Outcomes Among Women Who Used Intrapartum Water Immersion With No Complicating Factors

Labour interventions and outcomes for “low‐risk” women and their babies who used water immersion during labour or birth are presented in Table 2. Among the 73,229 women, 28.9% (*n* = 11,139) of nulliparous women and 4.9% (*n* = 1688) of parous women received obstetric or anesthetic care during the first and second stages of labour and an additional 10.7% (*n* = 2919) of primiparous and 5.0% (*n* = 1645) of parous women received obstetric care during or following the third stage of labour. Across the intrapartum episode 39.6% of primiparous and 9.9% of parous women who commenced labour without complications and under midwifery care, received obstetric or anesthetic care during the intrapartum period.

**TABLE 2:** Intrapartum events, interventions, and neonatal outcomes among women who used water immersion without recorded “risk‐factors” at water entry.

Outcome (study population, source)	Nulliparous (parity 0)	Multiparous (parity 1–3)	Total
Women using water immersion without recorded “risk factors” at water entry (MIS)	*N* = 38,525	*N* = 34,704	*N* = 73,229
Transfer to obstetric or anesthetic care during 1st or 2nd stage of labour	11,139 (28.9)	1688 (4.9)	12,827 (17.5)
Labour augmentation with oxytocin	3279 (8.5)	215 (0.6)	3494 (4.8)
Complex analgesia or anesthesia (epidural, spinal, general anesthetic, IV Remifentanil, pudendal block)	7395 (19.2)	972 (2.8)	8367 (11.4)
Pyrexia/suspected infection with investigation/treatment	756 (2.0)	102 (0.3)	858 (1.2)
Cardiotocograph described as pathological/suspicious/abnormal/non‐reassuring	5037 (13.1)	787 (2.3)	5824 (8.0)
Breech identified during labour	52 (0.1)	7 (0.02)	59 (0.1)
Mode of birth (MIS)			
Spontaneous vaginal birth	30,056 (78.0)	33,871 (97.6)	63,927 (87.3)
Forceps	3830 (9.9)	304 (0.9)	4134 (5.6)
Ventouse (inc. other vacuum)	2315 (6.0)	239 (0.7)	2554 (3.5)
Emergency caesarean section	2289 (5.9)	252 (0.7)	2541 (3.5)
Vaginal breech birth	29 (0.08)	36 (0.1)	65 (0.09)
Elective caesarean section	6 (0.02)	0 (0.0)	6 (0.008)
Not recorded	0	2	2
Shoulder dystocia (MIS)	623 (1.6)	635 (1.8)	1258 (1.7)
Postpartum interventions and outcomes (among all women using water immersion without recorded risk factors at water entry) (MIS)			
Recorded interventions following birth	7207 (18.7)	2081 (6.0)	9288 (12.7)
IV therapy (oxytocin, blood transfusion, plasma expanders, other IV fluids)	5654 (14.7)	1492 (4.3)	7146 (9.8)
Blood transfusions	161 (0.4)	62 (0.2)	223 (0.3)
Manual removal of placenta	1053 (2.7)	613 (1.8)	1666 (2.3)
OASI repair by mode of birth			
All modes of birth (inc. caesarean)	1954 (5.1)	463 (1.3)	2417 (3.3)
Spontaneous Vaginal Birth	1503 (5.0)	438 (1.3)	1941 (3.0)
Forceps	367 (9.6)	20 (6.6)	387 (9.4)
Ventouse (inc. other vacuum)	83 (3.6)	5 (2.1)	88 (3.4)
Outcomes following birth			
Postpartum sepsis	15 (0.04)	5 (0.01)	20 (0.03)
Postpartum haemorrhage^a^			
Recorded blood loss ≥ 500 mL	10,039 (26.1)	4186 (12.1)	14,225 (19.4)
Recorded blood loss ≥ 1000 mL	2492 (6.5)	991 (2.9)	3483 (4.8)
Recorded blood loss ≥ 1500 mL	877 (2.3)	377 (1.1)	1254 (1.7)
Treatment in women with blood loss ≥ 500 mL (categories not mutually exclusive)	*N* = 10,039	*N* = 4186	*N* = 14,225
Any intravenous therapy	5628 (56.1)	1487 (35.5)	7115 (50.0)
Intravenous fluids (inc. NaCl, Hartmann's, normal saline)	3934 (39.2)	980 (23.4)	4914 (34.5)
Oxytocin infusion	3715 (37.0)	1071 (25.6)	4786 (33.6)
Blood transfusion/products	134 (1.4)	54 (1.3)	188 (1.3)
Plasma expanders (inc. Plasma‐Lyte, Gelofusion, Colloids)	762 (7.6)	207 (4.9)	969 (6.8)
Postpartum complications (among women using water immersion without recorded risk factors at water entry who remained under midwifery care for birth) (MIS)	*N* = 27,386	*N* = 33,016	*N* = 60,402
Transfer/referral to obstetric care during or following 3rd stage of labour	2919 (10.7)	1645 (5.0)	4564 (7.6)
Infant outcomes			
Administration of intravenous antibiotics commenced within 48 h of birth^b^ (P, MIS)	473/9138 (5.2)	196/9211 (2.1)	669/18,349 (3.7)
Neonatal unit admission with respiratory support (P, NNRD)	203/10,017 (2.0)	117/9902 (1.2)	320/19,919 (1.6)
Secondary infant outcomes where collected across whole study period	*N* = 38,525	*N* = 34,704	*N* = 73,229
Administration of intravenous antibiotics commenced within 48 h of birth^b^ (W, NNRD)	1414/34,072 (4.2)	614/31,012 (2.0)	2028/64,919 (3.1)
Neonatal unit admission with respiratory support (W, NNRD)	620 (1.6)	336 (1.0)	956 (1.3)
Intrapartum or neonatal death (W, MIS/NNRD)	13 (0.34 per 1000 births)	12 (0.35 per 1000 births)	25 (0.34 per 1000 births)

*Note:* All data presented as *n* (%).

Abbreviations: MIS, Maternity Information System; NNRD, National Neonatal Research Database; OASI, obstetric anal sphincter injury; P, prospective data collection only; W, whole data collection.

^a^ Postpartum hemorrhage is determined from the total blood loss recorded at/after delivery.

^b^ Excludes four sites that did not record any postnatal outcome.

Primiparous women, compared to parous women, had higher rates of labour interventions including: augmentation of labour with Oxytocin (8.5% vs. 0.6%); caesarean section (5.9% vs. 0.7%); birth assisted with forceps (9.9% vs. 0.9%); or ventouse (6.0% vs. 0.7%); manual removal of placenta (2.7% vs. 1.8%); a higher rate of recorded blood loss ≥ 1000 mL (6.5 vs. 2.9%) and similar rates of blood transfusion (1.4% vs. 1.3%). Rates of administration of antibiotics to neonates within 48 h of birth were higher among babies born to nulliparous women compared to parous women (4.2% vs. 2.0%).

### 4.3 Management of the Third Stage of Labour Among “Low Risk” Women Who Used Water Immersion and Remained Under Midwifery Care for Birth

Among women who used intrapartum water immersion, physiological management of the third stage of labour was more frequent following waterbirth than following births out of water, 27.1% versus 10.9% respectively (Table 3). The rate of postpartum transfer to obstetric care following waterbirth, including treatment for hemorrhage, manual removal of placenta, or perineal repair, was 6.5% following waterbirths and 9.6% following births out of water (Table 3).

**TABLE 3:** Management of the third stage of labour by births in and out of water following uncomplicated labour.

Outcome (study population, source)	Waterbirth *N* = 39,627	Birth out of water *N* = 20,775
Active versus physiological management (MIS)		
Active management^a^	28,855 (72.9)	18,497 (89.1)
Physiological management	10,737 (27.1)	2260 (10.9)
Management unknown	35	18
Referral to obstetric care during or after 3rd stage of labour (MIS)		
No obstetric referral	37,063 (93.5)	18,775 (90.4)
Obstetric referral and interventions	2564 (6.5)	2000 (9.6)
Reason for obstetric involvement during or after third stage (categories not mutually exclusive)		
Treatment for hemorrhage	1591 (4.0)	1289 (6.2)
Manual removal of placenta	710 (1.8)	518 (2.5)
OASI repair	999 (2.5)	785 (3.8)
Sepsis	2 (0.0)	4 (0.0)
Postpartum haemorrhage^b^		
Recorded blood loss ≥ 500 mL	5199 (13.1)	3329 (16.0)
Blood loss ≥ 1000 mL	1165 (2.9)	797 (3.8)
Blood loss ≥ 1500 mL	445 (1.1)	274 (1.3)
Treatment for hemorrhage in women with recorded blood loss ≥ 500 mL	*N* = 5199	*N* = 3329
Intravenous therapy (categories not mutually exclusive)	1579 (30.4)	1282 (38.5)
Intravenous fluids (inc. NaCl, Hartmann's solution)	1090 (21.0)	902 (27.1)
Oxytocin infusion	987 (19.0)	742 (22.3)
Blood transfusion/blood products	75 (1.4)	43 (1.3)
Plasma expanders (inc. Plasma‐Lyte, Gelofune Colloids)	240 (4.6)	167 (5.0)

*Note:* All data presented as *n* (%).

Abbreviations: MIS, Maternity Information System; OASI, obstetric anal sphincter injury.

^a^ As coded by the midwife in the birth record.

^b^ Postpartum hemorrhage was determined from the estimated or measured recorded blood loss at/after delivery.

Data were available for 10,760 waterbirths on whether the placenta was delivered in water. Delivery of the placenta in water varied across sites from 0.6% to 29.3%, with no association between this and the number of waterbirths being performed at sites (Figure S4).

Among the 926 births at which the placenta was delivered in water, physiological management of the third stage was recorded in 60.3% (*n* = 558) of cases and active management in 39.7% (*n* = 368) of cases (Table 4). Where the placenta was delivered out of water, active management was recorded in 82.2% (*n* = 7134) of cases.

**TABLE 4:** Management of the third stage of labour and rate of postpartum hemorrhage for “low‐risk” women following waterbirth.

	All women		Nulliparous women		Multiparous women	
	Placenta delivered		Placenta delivered		Placenta delivered	
	In water	Out of water	In water	Out of water	In water	Out of water
Total women	926	8679	321	3161	605	5518
Management of the third stage of labour						
Active management	368 (39.7)	7134 (82.2)	110 (34.3)	2579 (81.6)	258 (42.6)	4555 (82.5)
Physiological management	558 (60.3)	1544 (17.8)	211 (65.7)	52 (18.4)	347 (57.4)	962 (17.5)
Management not recorded	0	1	0	0	0	0
Postpartum haemorrhage^a^						
Recorded blood loss ≥ 500 mL	109 (11.8)	1195 (13.8)	53 (16.5)	569 (18.0)	56 (9.3)	626 (11.3)
Recorded blood loss ≥ 1000 mL	18 (1.9)	278 (3.2)	8 (2.5)	128 (4.0)	10 (1.7)	150 (2.7)
Recorded blood loss ≥ 1500 mL	6 (0.6)	98 (1.1)	2 (0.8)	46 (1.5)	4 (0.7)	52 (0.9)
Treatment for hemorrhage in women with blood loss ≥ 500 mL (not mutually exclusive)	*N* = 109	*N* = 1195	*N* = 53	*N* = 569	*N* = 56	*N* = 626
Any intravenous therapy	22 (20.2)	440 (32.8)	12 (22.6)	201 (35.3)	10 (17.9)	182 (29.1)
Intravenous fluids (inc. NaCl, Hartmann's solution)	16 (14.7)	301 (2.4)	10 (18.9)	147 (25.8)	6 (10.7)	109 (17.4)
Oxytocin infusion	15 (13.8)	281 (20.9)	5 (1.0)	109 (19.2)	10 (17.9)	137 (21.9)
Plasma expanders	2 (1.8)	58 (4.3)	1 (1.9)	23 (4.0)	1 (1.8)	28 (4.5)
Blood transfusion/products	0 (0.0)	25 (1.9)	0 (0.0)	14 (2.5)	0 (0.0)	9 (1.4)

*Note:* All data presented as *n* (%).

^a^ Postpartum hemorrhage was determined from estimated or measured recorded blood loss at/after delivery.

Following waterbirth, rates of recorded blood loss of ≥ 500 mL were similar for women who delivered their placenta in water compared to those leaving the water during the third stage, 11.8% and 13.7% respectively. Among women with a recorded blood loss of ≥ 500 mL following waterbirth, receipt of intravenous fluids was higher for women who delivered their placenta in water compared to those leaving the water during the third stage (14.7% vs. 2.4%), whereas use of an oxytocin infusion was lower (13.8% vs. 20.9%). The rate of recorded PPH of ≥ 1000 mL was 1.9% among women who delivered their placenta in water and 3.3% among women who left the water during the third stage, with no evidence of a significant increase (adjusted odds ratio 0.70, 2‐sided 95% CI, 0.42 to 1.18, *p*‐value 0.181). Among the 926 women who, following waterbirth, also delivered their placenta in water, none received a blood transfusion or blood products, compared to 1.9% of women who had a waterbirth then left the water during the third stage of labour.

## 5 Discussion

To our knowledge, this was the largest global study of intrapartum water immersion to date.

The finding of a reduction in water use and water births over the study period is primarily considered to have been influenced by increasing proportions of women experiencing induction of labour or elective caesarean section as respective rates in England increased between 2015 and 2022 from 27% to 33%, and from 14% to 23% [16]. This reflects changes in UK maternity care over the study period including national policy on stillbirth reduction lowering thresholds to artificially end pregnancies at term [17], repercussions of investigations into maternity services in various locations in the UK following reports of clusters of avoidable perinatal deaths in midwifery and obstetric settings [18, 19] and sustained criticism and undermining of the credibility of midwifery care in the press and social media [20]. The decline in the use of water for labour and birth found during Covid‐19 restrictions was more modest than anticipated. Many midwifery units were reconfigured at the start of the pandemic [21] and advice was against the use of water immersion for any women who may have Covid‐19 [22]. In contrast, it was recognized that home births could reduce pressure on hospitals [23] and some sites reported that more women opted for a home birth to avoid hospital admission with associated infection risk and limitation on partner attendance.

Reflecting the findings of qualitative work undertaken during the study [24] site and demographic disparities in the use of water immersion during labor were found. Some of the observed differences will reflect clinical need, including parous women with obstetric risk factors from previous births, but differences may also reflect bias in the offer [25] or different levels of knowledge of the option of water immersion resulting in inequitable access. These findings were similar to previous UK studies [26, 27] that identified women from non‐white ethnic groups and women living with social disadvantage experienced limitations in the offer of birth choices, including assumptions by midwives that women from ethnic minority communities are unfamiliar with waterbirth or find it undesirable [25].

Rates of modes of birth were similar to those found among “low risk” women planning birth in midwifery settings in the UK between 2000 and 2010 [7, 28]. The similarity in birth outcomes over time period is important as women considering their birth options should be provided with contemporary and geographically relevant information. The UK Royal College of Obstetricians and Gynecologists recommend that women planning a vaginal birth should be informed that combined rates of instrumental and emergency caesarean birth are around 88% among nulliparous women and around 32% among parous women [29]. This fails to take account of the consistent low interventions rates among women planning birth outside of obstetric units, and may result in more women choosing a caesarean section to avoid, what they have been informed will be, a high chance of instrumental or operative birth.

To our knowledge, this is the first large study to report outcomes associated with placental delivery into water. As it is recommended that controlled cord traction and placental delivery with active management of the third stage of labour occur after the woman has left the water [30] the finding that 39.7% of women who delivered the placenta in water received active third stage management was unexpected. Our results provide reassurance that following waterbirth, placental delivery into water is not associated with increased risk of severe hemorrhage, and it facilitates skin‐to‐skin contact [31] often disrupted during pool exit.

### 5.1 Strengths and Weaknesses

Strengths and limitations of the POOL cohort study are described fully elsewhere [5]. Strengths include the large sample size, the minimization of bias and absence of self‐selection bias from the model of non‐consent, and the ability to control for a range of confounders.

The study has several limitations, including the nature of routinely collected clinical data, including the inability to check the validity of outlying data entries and it being underpowered to draw conclusions on rare but important outcomes such as perinatal death. The potential also remains that the use of water immersion during labor was not captured in the electronic records of individual women, excluding them inappropriately. Despite the availability of training and guidance on blood loss during waterbirth [32], it remains that this can be expected to be less accurate than blood loss recorded in births out of water, particularly in units where blood loss measurement by weighting has been implemented [33]. Also, unexplored confounders may have influenced study findings, and it remains possible that women in the various study groups may have differed in ways that may be associated with differences in outcomes. For example, women giving birth and delivering the placenta in water may have different attitudes towards labor and birth from women who chose to leave the water before birth.

The findings, as with other analyses from the POOL study, describe outcomes relating to NHS maternity care between 2015 and 2022. Over the study period thresholds for the offer of induction of labour and other intrapartum interventions have changed and the proportion of women commencing spontaneous labour decreased [34]. It is possible that in the future women using water for labour and birth will have different characteristics, and midwives less experience of water birth than those included in our study.

## 6 Conclusion

This large UK study of water immersion during labour and birth provides important information for policy makers, maternity health professionals, and for women and families considering the option of intrapartum water immersion. Care providers need to ensure equal access to intrapartum water immersion across demographic groups and provide women with evidence‐based information on water immersion analgesia, waterbirth, and third stage management, which takes account of their risk status and birth choices.

## Ethics Statement

Research Ethics Committee (REC) approval was granted by the REC for Wales (18/WA/0291) and the transfer and use of identifiable data was approved by the Health Research Authority (HRA) Confidentiality Advisory Group (CAG) (18/CAG/0153).

## Consent

No consent was required from participants, but women in the prospective cohort could request not to be included in the study.

## Conflicts of Interest

All authors have completed the ICMJE uniform disclosure form and declare: the NIHR provided funding to the employing institutions of J.S., C.B., P.B., R.C.‐J., S.C., J.C., B.H., F.L.‐W., S.M., S.P., and M.R. to support the undertaking of the submitted work; C.G. received salary support from the Medical Research Council paid to his institution through a Personal Fellowship (Clinician Scientist and Transition Support); no financial relationships with any organizations that might have an interest in the submitted work in the previous three years; no other relationships or activities that could appear to have influenced the submitted work.

## Supporting information


Data S1.

## Data Availability

Applicants interested in requesting data for non‐commercial purposes should apply via the data application form available on the Centre for Trials Research website (https://www.cardiff.ac.uk/centre‐for‐trials‐research/collaborate‐with‐us/data‐requests). Following an internal and peer review of the application and subject to approval, data may be released under a data transfer agreement.  In the first instance, enquiries about access to the data should be addressed to Professor Julia Sanders, School of Healthcare Sciences, Cardiff University.   For the purpose of open access, the author has applied a ‘Creative Commons Attribution (CC BY) licence to any Author Accepted Manuscript (AAM) version arising’.
