# Supplementary material for: Characteristics of Women, Intrapartum Interventions, and Maternal and Neonatal Outcomes Among Users of Intrapartum Water Immersion: The UK POOL Cohort Study
Source: Birth. 2025 May 12;53(1):55–66. doi: 10.1111/birt.12921 (PMC12894509; doi:10.1111/birt.12921)
Supplement: Supplementary file 1 — Data S1. [file BIRT-53-55-s001.docx]

# Supplementary material

### **Participating study sites**

**Figure S1**


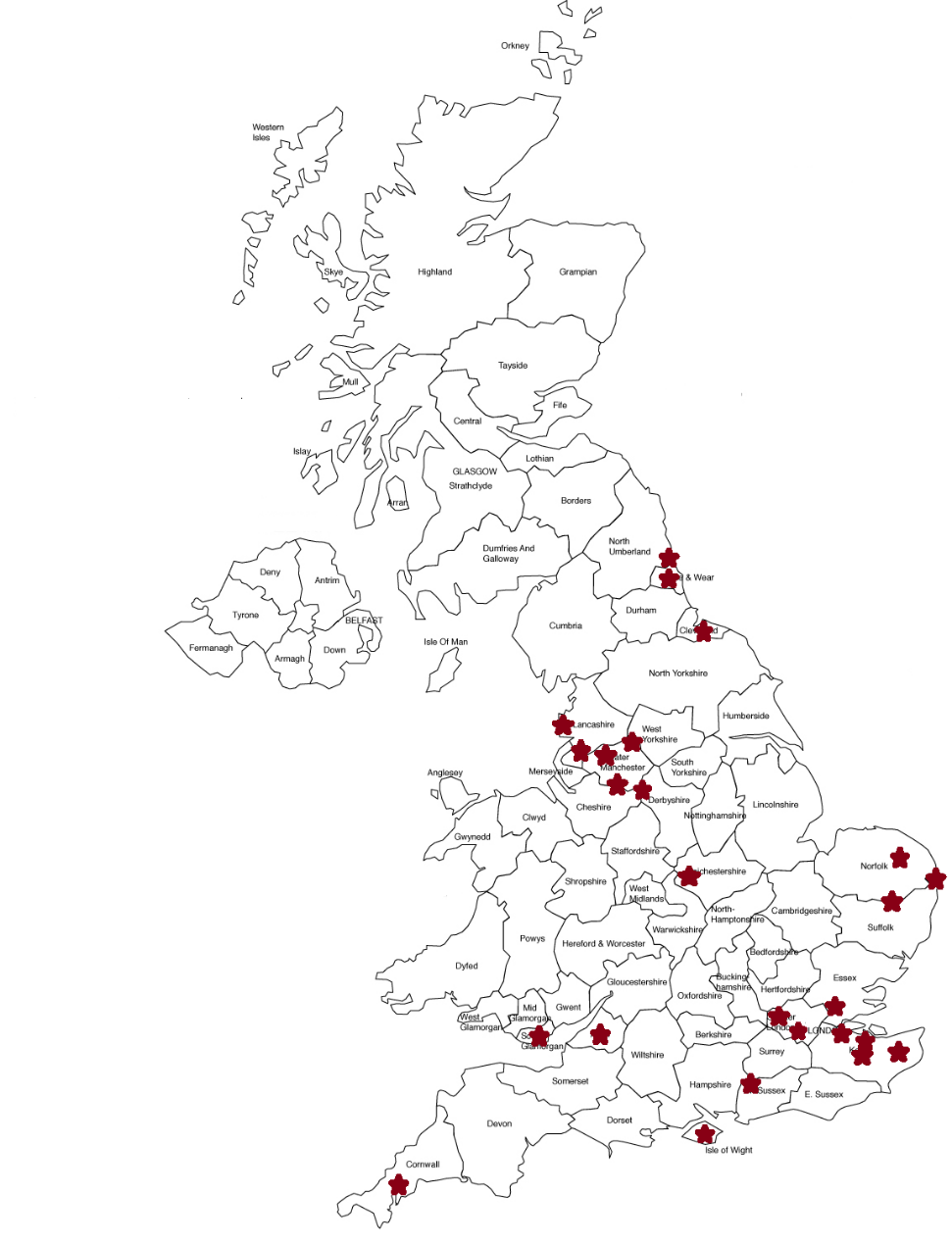


### **NICE Intrapartum Care Guidelines**

Table S1.1 and S1.2 show extracts from NICE Guidance^[[1]](#footnote-1)^ providing medical conditions or situations in which there is increased risk for the woman or baby during or shortly after labour, where care in an obstetric unit would be expected to reduce this risk. The factors listed in Table S1.3 and S1.4 are not reasons in themselves for advising birth within an obstetric unit, but indicate that further consideration of birth setting may be required.

Table S1.1 Medical conditions indicating increased risk suggesting planned birth at an obstetric unit

| **Disease area** | **Medical condition** |
| --- | --- |
| Cardiovascular | - Confirmed cardiac disease - Hypertensive disorders |
| Respiratory | - Asthma requiring an increase in treatment or hospital treatment - Cystic fibrosis |
| Haematological | - Haemoglobinopathies – sickle‑cell disease, beta‑thalassaemia major - History of thromboembolic disorders - Immune thrombocytopenia purpura or other platelet disorder or platelet count below 100×10^9^/litre - Von Willebrand's disease - Bleeding disorder in the woman or unborn baby - Atypical antibodies which carry a risk of haemolytic disease of the newborn |
| Endocrine | - Hyperthyroidism - Diabetes |
| Infective | - Risk factors associated with group B streptococcus whereby antibiotics in labour would be recommended - Hepatitis B/C with abnormal liver function tests - Carrier of/infected with HIV - Toxoplasmosis – women receiving treatment - Current active infection of chicken pox/rubella/genital herpes in the woman or baby - Tuberculosis under treatment |
| Immune | - Systemic lupus erythematosus - Scleroderma |
| Renal | - Abnormal renal function - Renal disease requiring supervision by a renal specialist |
| Neurological | - Epilepsy - Myasthenia gravis - Previous cerebrovascular accident |
| Gastrointestinal | - Liver disease associated with current abnormal liver function tests |
| Psychiatric | - Psychiatric disorder requiring current inpatient care |

Table S1.2 Other factors indicating increased risk suggesting planned birth at an obstetric unit

| **Factor** | **Additional information** |
| --- | --- |
| Previous complications | - Unexplained stillbirth/neonatal death or previous death related to intrapartum difficulty - Previous baby with neonatal encephalopathy - Pre‑eclampsia requiring preterm birth - Placental abruption with adverse outcome - Eclampsia - Uterine rupture - Primary postpartum haemorrhage requiring additional treatment or blood transfusion - Retained placenta requiring manual removal in theatre - Caesarean section - Shoulder dystocia |
| Current pregnancy | - Multiple birth - Placenta praevia - Pre‑eclampsia or pregnancy‑induced hypertension - Preterm labour or preterm prelabour rupture of membranes - Placental abruption - Anaemia – haemoglobin less than 85 g/litre at onset of labour - Confirmed intrauterine death - Induction of labour - Substance misuse - Alcohol dependency requiring assessment or treatment - Onset of gestational diabetes - Malpresentation – breech or transverse lie - BMI at booking of greater than 35 kg/m2 - Recurrent antepartum haemorrhage - Small for gestational age in this pregnancy (less than fifth centile or reduced growth velocity on ultrasound) - Abnormal fetal heart rate/doppler studies - Ultrasound diagnosis of oligo‑/polyhydramnios - Cholestasis* - Labour outside of 37+0 and 41+6* |
| Previous gynaecological history | - Myomectomy - Hysterotomy |

*Some additional conditions, not included in the NICE guidelines, have been identified that if present would be also regarded as contraindications to water immersion in labour and therefore if present would classify the woman as ‘high-risk’

Table S1.3 Medical conditions indicating individual assessment when planning place of birth

| **Disease area** | **Medical condition** |
| --- | --- |
| Cardiovascular | - Cardiac disease without intrapartum implications |
| Haematological | - Atypical antibodies not putting the baby at risk of haemolytic disease - Sickle‑cell trait - Thalassaemia trait - Anaemia – haemoglobin 85–105 g/litre at onset of labour |
| Infective | - Hepatitis B/C with normal liver function tests |
| Immune | - Non‑specific connective tissue disorders |
| Endocrine | - Unstable hypothyroidism such that a change in treatment is required |
| Skeletal/neurological | - Spinal abnormalities - Previous fractured pelvis - Neurological deficits |

Table S1.4 Other factors indicating individual assessment when planning place of birth

| **Factor** | **Additional information** |
| --- | --- |
| Previous complications | - Stillbirth/neonatal death with a known non‑recurrent cause - Pre‑eclampsia developing at term - Placental abruption with good outcome - History of previous baby more than 4.5 kg - Extensive vaginal, cervical, or third‑ or fourth‑degree perineal trauma - Previous term baby with jaundice requiring exchange transfusion |
| Current pregnancy | - Antepartum bleeding of unknown origin (single episode after 24   weeks of gestation)   - BMI at booking of 30–35 kg/m2 - Blood pressure of 140 mmHg systolic or 90 mmHg diastolic or   more on 2 occasions   - Clinical or ultrasound suspicion of macrosomia - Para 4 or more - Recreational drug use - Under current outpatient psychiatric care - Age over 35 at booking |
| Fetal indications | - Fetal abnormality |
| Previous gynaecological history | - Major gynaecological surgery - Cone biopsy or large loop excision of the transformation zone - Fibroids |

### **Study flow chart**

**Figure S2**


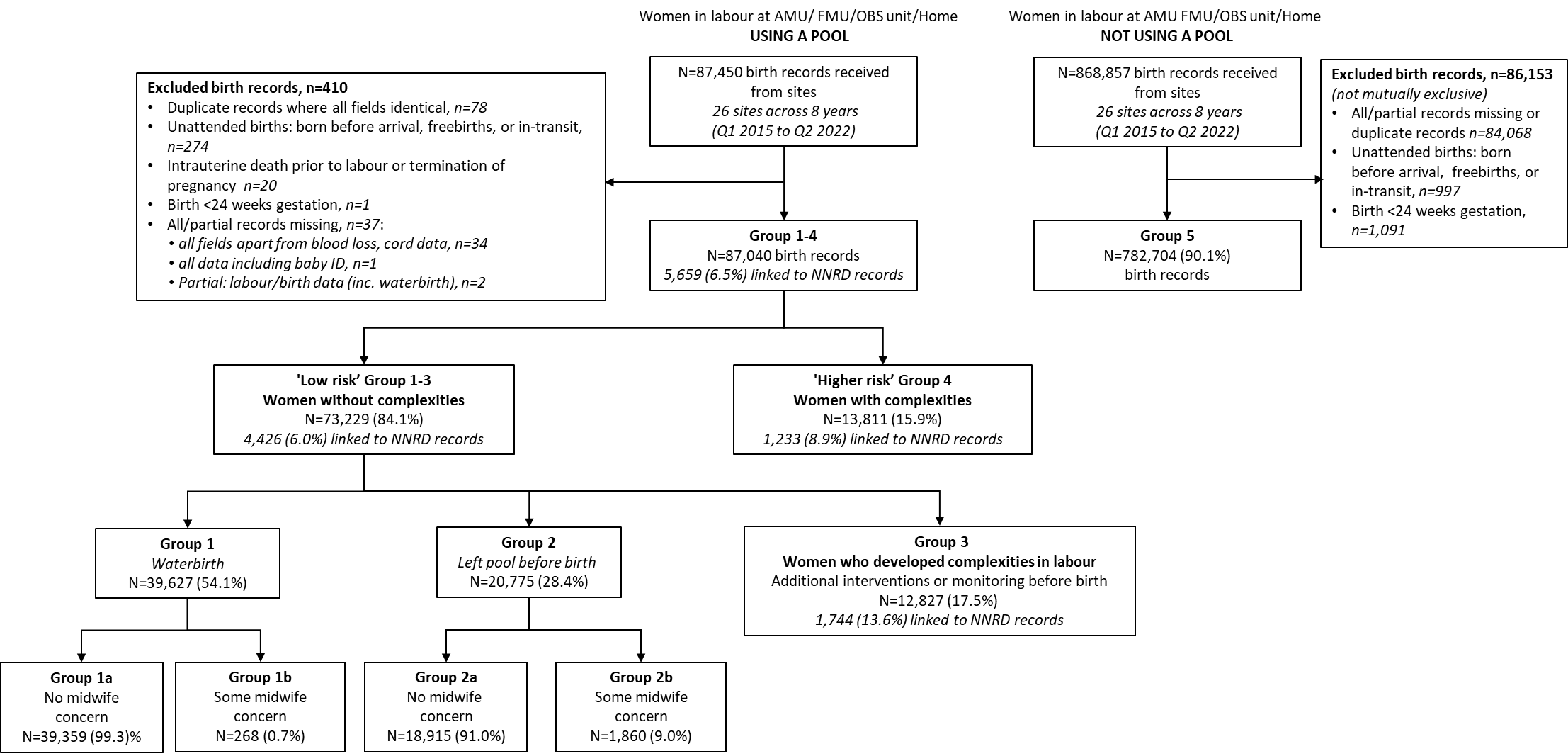


**Figure S3:** Rates of water immersion and waterbirth (based on all births) by site


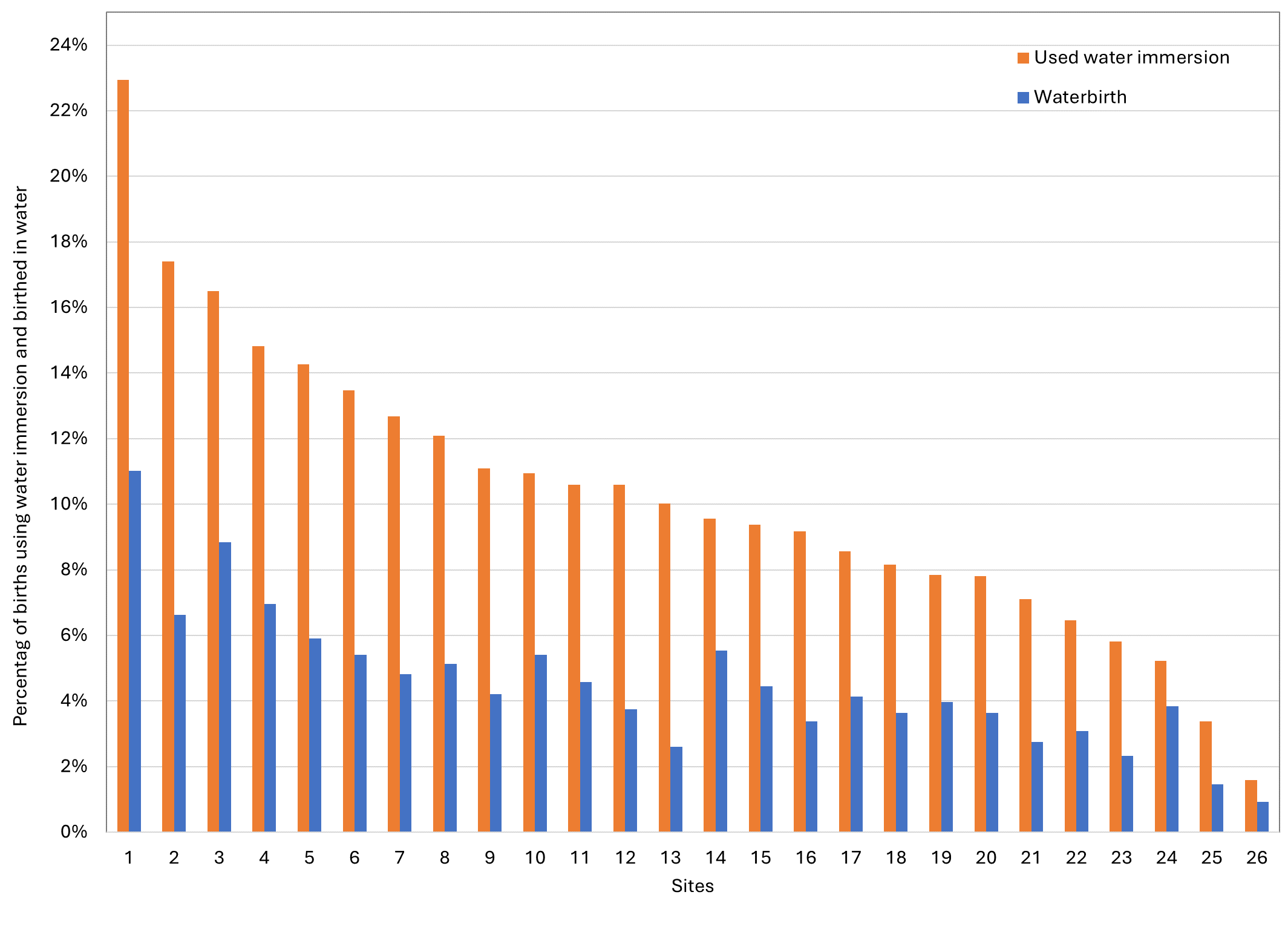


For n=26 sites; n=869,744 birth records; n=87,040 using water immersion; 39,627 waterbirths.
Site numbers based on descending rates of water immersion.

**Figure S4**: Site variation in for ‘low-risk’ women who, following birth in water, delivered the placenta underwater or left the water prior to delivery of the placenta


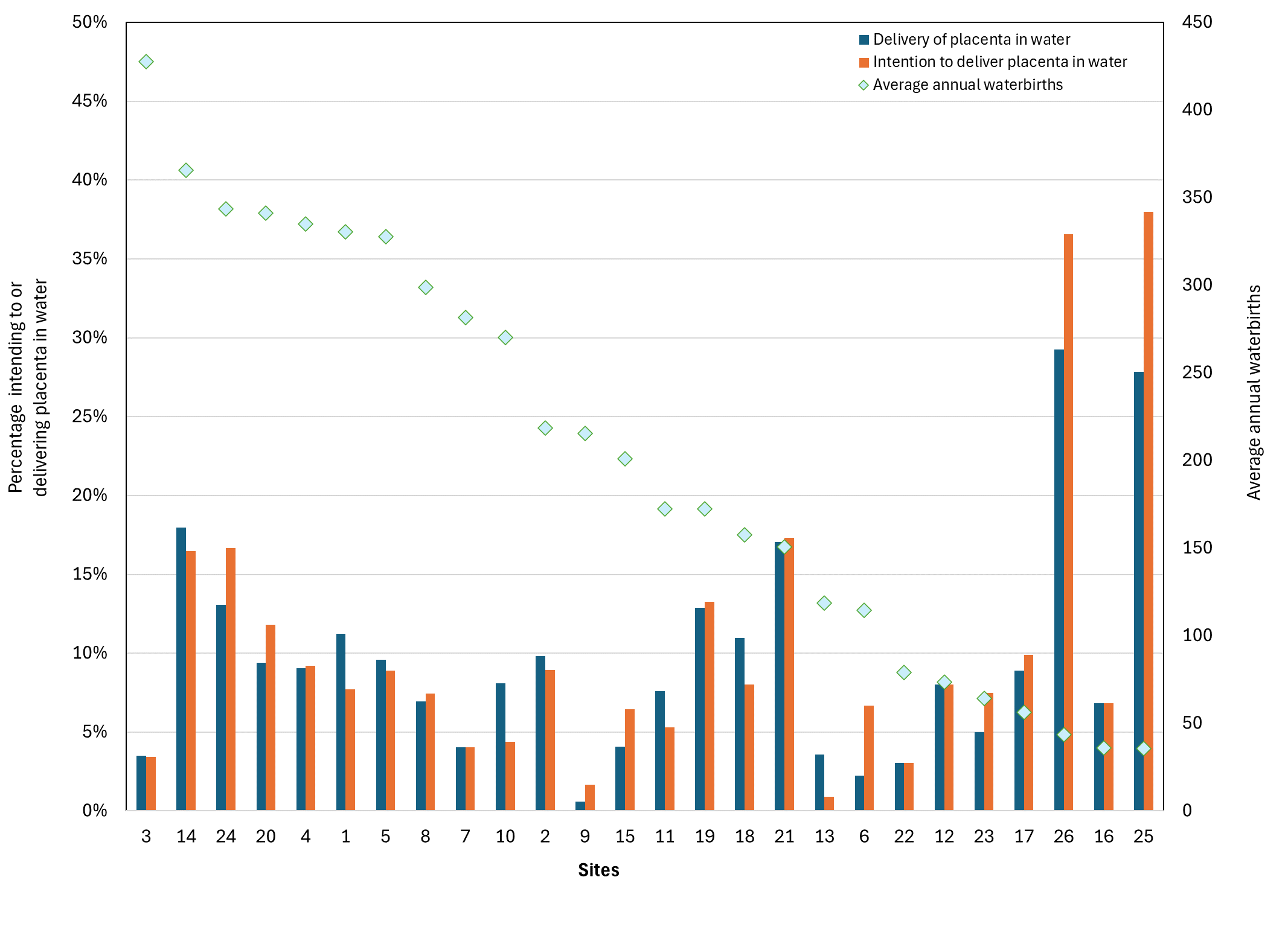


Site numbers based on descending rates of water immersion (as seen in Figure S2).

1. National Institute for Health and Care Excellence. Intrapartum Care: Care of healthy women and their babies during childbirth. London: RCOG Press, 2023. [↑](#footnote-ref-1)
